# Food acculturation and nutrient intake among international students in India: an exploratory cross-cultural study

**DOI:** 10.3389/fnut.2026.1865569

**Published:** 2026-08-07

**Authors:** Anuja Phalle, Devaki Gokhale, Burhan Khaleel, Nusaiba Maiyyaya

**Affiliations:** Department of Nutrition & Dietetics, Symbiosis School of Culinary Arts & Nutritional Sciences, Symbiosis International (Deemed) University, Pune, Maharashtra, India

**Keywords:** dietary acculturation, dietary displacements, food acculturation, food insecurity, health trajectories, international students, migration, socio-cultural factors

## Abstract

**Introduction:**

Food acculturation and dietary transitions among international students (IS) in Western countries have been reported to result in undesirable changes in eating behaviors and dietary patterns, which may negatively affect nutritional status and health after migration. These dietary changes may be influenced by various factors specific to the host country. Given the recent increase in IS enrollment in culturally diverse Asian countries such as India, there is a need to investigate food acculturation in the Indian context. Therefore, this study explored food acculturation, changes in eating behaviors, the factors influencing these changes, and pre- and post-migration differences in anthropometric measures and nutrient intakes.

**Methods:**

In this exploratory study with a retrospective pre–post design, IS (*n* = 320) enrolled at several universities in Pune, India, an education hub, were recruited through purposive sampling. An 83-item structured questionnaire with five sections: (a) sociodemographic information, (b) anthropometric parameters, (c) perceived factors influencing food acculturation, (d) changes in eating behaviors, and (e) food frequency questionnaire (FFQ), was developed, validated, and administered to capture pre- and post-migration changes. Portion sizes and FFQ data were converted to daily intakes to calculate nutrient intake using DietCal version 15.1.3. Multinomial regression analysis and the Wilcoxon signed-rank test were performed using Statistical Package for the Social Sciences (SPSS) version 30.0. Statistical significance was set at *p* ≤ 0.05.

**Results:**

IS (*n* = 320) with a mean age of 22.5 ± 4.1 years, height of 169.7 ± 10.3 cm, weight (pre-migration = 65.2 ± 14.3 *vs*. post-migration = 67.0 ± 14.1 kg), and body mass index (BMI) (pre-migration = 22.58 ± 4.1 *vs*. post-migration = 23.18 ± 3.9 kg/m^2^) participated in this study. The study participants reported increased meal skipping (78.7%), consumption of convenience foods (50.3%), eating out (50%), appetite loss (47.5%), replacing meals with snacks (54%), decreased portion size (43%), and a drop in meal frequency to two meals per day (40%) post-migration. These eating behaviors were significantly associated with age, sex, marital status, standard of living, education, region of origin, and Hindi-speaking ability for food purchases (all *p* < 0.05). Among the perceived factors, the majority reported past acculturation, food availability, accessibility, environment, affordability, sociocultural factors, religious factors, and culinary factors. Regarding food consumption frequency, a statistically significant (all *p* < 0.005) decrease was noted for beef, mutton, fish, seafood, eggs and poultry, milk and milk products, pulses and legumes, nuts and oilseeds, green leafy vegetables, and fruits. This low food intake was also reflected in nutrient intake, with declines (all *p* < 0.05) in protein, iron, calcium, vitamin B12, and zinc, whereas high energy, carbohydrate, and fat intakes were evidenced by increased body weight across regions of origin.

**Conclusion:**

Food acculturation patterns observed among IS led to undesirable changes in food and nutrient intake, which may have negative long-term health implications. Planning nutrition interventions that promote the adoption of local, nutritious food is of key importance, while conducting in-depth, qualitative, and longitudinal studies to achieve a better understanding of food acculturation in the Indian context is also needed.

## Introduction

Globalized education systems have accelerated educational tourism, leading to increased migration of international students (IS) in search of better opportunities and higher-quality education. Although Western countries have been the primary choice and host a large share of the 2.9 million IS (1), Asian countries, including India, have also become new destinations for educational tourism, with an expected 8% increase in IS enrollment by 2026 (2). Following migration to the host country, according to Berry’s acculturation model, an individual undergoes “acculturation: a multifaceted bidirectional process primarily involving transition in sociocultural and psychological dimensions among many others (3).” One significant yet often overlooked aspect of this transition is food acculturation, defined as the process by which migrants adopt the dietary practices of the host country while retaining elements of their traditional diet (4). Food acculturation is shaped by factors such as food availability, affordability, cultural and religious practices, social influences, and access to cooking facilities (5, 6). Migration-related acculturation, particularly dietary transition, plays a crucial role in the overall wellbeing and social integration of IS, often serving as a coping mechanism for cultural adjustment (7). This process of food acculturation has often been stressful and complex due to various factors, primarily individual, sociocultural, and environmental factors (4). Adaptation to new dietary cultures and habits can be challenging for IS, increasing acculturation-related food stress. Additionally, IS face language and financial barriers, academic stress, limited healthcare facilities, lack of social support, and discrimination, which further increase acculturative stress (8).

**Figure 1:**
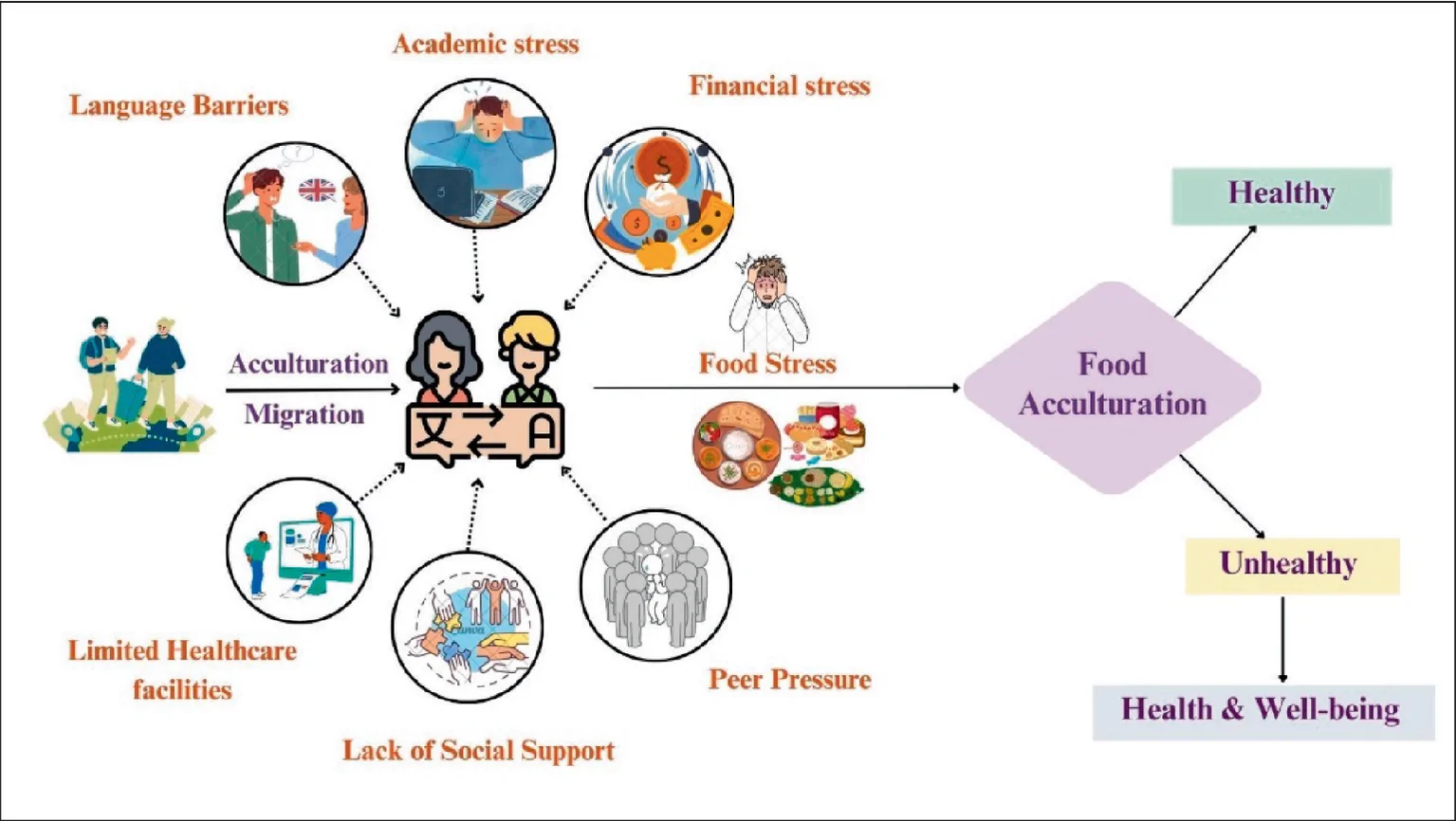
Schematic representation of the complex interplay between factors influencing food acculturation and its potential impact on the health and wellbeing of international students. **A**uthors’ own creation using Canva software.

All these factors may affect the pattern of food acculturation, leading to the adoption of healthy or unhealthy dietary patterns (see Figure 1). A study of South Asian immigrants in Canada reported the adoption of healthy cooking methods following migration (9). Unhealthy dietary patterns, such as under-eating nutritious foods or overconsumption of processed/ultraprocessed foods, have also been observed among IS. Increased meal skipping, changes in portion size, and adoption of high-fat, high-sugar Western dietary patterns have been observed among IS after migration (4, 10, 11). Moreover, unhealthy dietary adaptations may increase the risk of non-communicable diseases, as observed in previous cross-sectional studies reporting obesity among IS (7, 11–13). However, the long-term effects of migration on the health of IS remain poorly explored.

Food insecurity is a major challenge among IS, given their unique context, and can significantly alter their nutritional status, thereby influencing their health. IS may become more food insecure due to the financial burden associated with high tuition fees, living costs, and the unfamiliar food environment of the host country (14–16). Chronic food insecurity can create a state of nutritional insecurity among IS, as inadequate access to nutritious food may lead to the adoption of unhealthy dietary habits. Such a complex interplay of factors may have negative implications for the nutritional status, health, and overall wellbeing of IS (17).

In addition to providing better-quality education, internationalization produces a globally skilled and culturally competent workforce. Additionally, increased IS enrollment benefits the educational tourism industry and contributes to the nation’s economic capacity. The promotion of educational tourism necessitates the retention of IS, which requires comprehensive multidimensional support, including adequate food and nutrition support services, to safeguard their health and wellbeing in the host country. From this symbiotic partnership standpoint and the perspective of IS welfare, food-related acculturation experiences in the host country, changes in eating behaviors, and their impact on physical health become critical. Although global evidence provides a foundation for our understanding of food acculturation, a major research gap exists, highlighting the need for further research, particularly in culturally diverse, newly emerging education tourism destinations such as India. Therefore, in this exploratory study, a retrospective pre–post design was used to identify patterns of food acculturation, eating behaviors, and their sociodemographic and sociocultural factors among IS studying in Pune, India. Pune is a multicultural academic hub with a diverse international student population, providing a unique setting to examine these dynamics. Furthermore, differences in anthropometric parameters and nutrient intakes before and after migration were assessed by region of origin.

## Methods

### Participants and procedure

**Figure 2:**
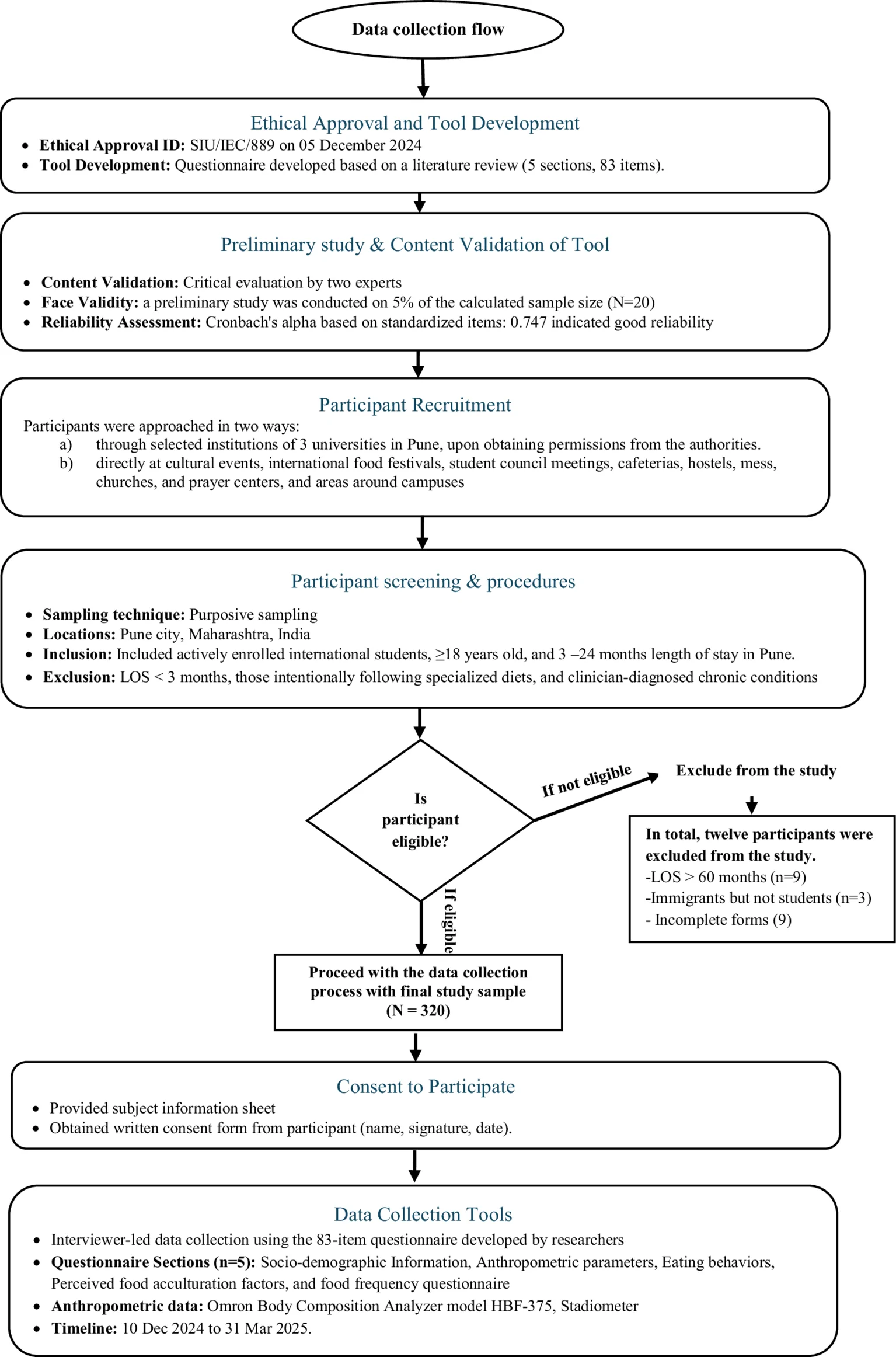
Data collection process flowchart.

An exploratory study with a retrospective pre–post design was conducted by recruiting international students, defined by the United Nations Educational, Scientific and Cultural Organization (UNESCO) (2023) as individuals aged 18–30 years who were studying in Pune, India, using purposive sampling (1). The exclusion criteria were as follows: length of stay (LOS) < 3 months; clinician-diagnosed medical conditions requiring a specialized diet; and intentional adherence to any dietary regimen. Based on the existing literature (4, 10, 18), the exclusion window of LOS < 3 months was selected because individuals tend to explore the new or unfamiliar foods in the host country during the first 3 months after migration, which could have affected the results. Three leading universities in Pune, India, were targeted for data collection due to their high international student enrollment. To capture retrospective data as accurately as possible based on participants’ recall, the pre-migration period was defined as the 1-month before migration, and the post-migration period was defined as 3–24 months after migration, during which data from the preceding month were collected. A systematic procedure, as depicted in Figure 2, was followed for data collection. This study was conducted in accordance with the Declaration of Helsinki and received approval from the Independent Ethics Committee (SIU/IEC/889) of Symbiosis International (Deemed) University. All participants were informed about the study through a participant information sheet, and those who voluntarily agreed to participate provided written consent. The questionnaire was administered in an interviewer-led format by researchers who measured anthropometric parameters and used standard food models to capture the food intake accurately.

Using Cochran’s formula, *n* = *Z*^2^ *p* × (1−*p*)/*d*^2^, a sample size of 267 was estimated with a population proportion (*p*) of 50%, a 95% confidence level, and a 6% margin of error (19). Given an attrition rate of 20%, a sample size of 320 was required; however, 341 participants responded to the survey. After excluding participants who did not meet the eligibility criteria (*n* = 12) and those with incomplete responses (*n* = 9), the final analysis was based on a sample of 320.

### Data collection tools

An 83-item questionnaire was developed by the researchers based on previous research on immigrant health, particularly among the international student population (3–11, 13, 15, 17, 20–22). The questionnaire consisted of five sections: Section A, Sociodemographic information; Section B, Anthropometric parameters; Section C, Perceived factors influencing food acculturation; Section D, Pre- and post-migration changes in eating behaviors; and Section E, Food frequency questionnaire. Two experts in the field reviewed the study questionnaire for content validity. Additionally, the face validity of the entire questionnaire was established through a preliminary study conducted with 5% of the estimated sample size (*N* = 20). The data from the preliminary study were excluded from the final analysis. The final questionnaire was modified for clarity, ease of understanding, reduced repetition, and reduced participant burden. The reliability analysis of the entire questionnaire showed acceptable reliability (Cronbach’s alpha based on standardized items: 0.747). The standardized Cronbach’s alpha was used due to differences in scale ranges across the questionnaire’s sections.

*Section A*: Sociodemographic information (10 items), including age, sex, marital status, standard of living, education, length of stay, region of origin, sponsorship, current living arrangement, and ability to speak Hindi for food purchase, was reported by the participants. *Section B*: Anthropometric parameters (three items), including height, weight, and body mass index (BMI), were collected by the researchers from participants’ health check-up records to obtain pre-migration data. These health checks were mandatory for participants before they joined the university. The post-migration weights were measured by researchers using a calibrated weighing scale and an Omron Body Composition Analyzer model HBF-375, and height was recorded using a stadiometer. *Section C*: Perceived factors influencing food acculturation had 9 subsections and 44 items, such as previous acculturation experience and present orientation (3 items), dietary changes post-migration (4 items), food availability (6 items), food accessibility (4 items), food affordability (3 items), physical health post-migration (4 items), social factors (5 items), religious factors (2 items), and culinary factors (13 items). Participants rated the items on a 5-point Likert scale ranging from “Strongly agree” (1) to “Strongly disagree” (5) for all the domains except for the “Culinary factors” domain, wherein a reverse 5-point Likert scale starting from “Never” (0), “Almost never” (1), “Sometimes” (2), “Fairly” (3), “Very often” (4) was used. *Section D*: Changes in eating behaviors (8 items), wherein both the pre- and post-migration data were self-reported; however, pre-migration changes were based on recall. This section recorded the eating behaviors, such as a change in diet (yes/no), number of meals/day (2/3/>4 meals), meal skipping (yes/no), type of meal skipped (breakfast/lunch/dinner), and appetite loss (yes/no). For eating behaviors, such as portion size, consumption of convenience foods, eating out, and replacing meals with snacks, participants were required to self-evaluate and report changes as increased, decreased, or unchanged. *Section E*: Food frequency questionnaire (FFQ), consisting of 20 food groups, reported with frequencies such as 1 = twice daily, 2 = daily, 3 = 2–3 times per week, 4 = weekly, 5 = twice monthly, 6 = monthly, and 7 = never, was developed and modified based on the USDA food classification system (23) to include major food groups, given the diverse regions of origin of the study population. The frequency of food consumption in the region of origin during the month before migration was based on recall. To capture post-migration data, participants also recalled and self-reported the frequency of food consumption during the month before data collection. The food groups included cereal group A (e.g., wheat, bread, naan, and pasta), cereal group B (e.g., rice), beef, mutton/lamb, fish, seafood (e.g., crabs and oysters), poultry and eggs, pulses and legumes, nuts and oilseeds, milk, and milk products (e.g., curd, buttermilk, and yogurt). Vegetable groups included group A (fruit vegetables; e.g., ash gourd, bitter gourd, brinjal, capsicum, cucumber, cauliflower, and zucchini), group B (roots and tubers; e.g., potato and radish), and group C (green leafy vegetables). Fruit groups included common fruits (e.g., apple and banana) and exotic or seasonal fruits (e.g., kiwi, dragon fruit, and berries). Sugars and sweets, fat/oil, convenience/processed foods (e.g., chips, instant noodles, ready-to-eat foods), and dietary supplements were also included. Additionally, participants were required to report portion sizes for each food group at the reported frequency. To estimate portion sizes accurately, trained researchers showed participants standard food pictures and models, along with measuring cups and spoons, and asked them to report portion sizes in household measures, indicating small, medium, or large portions (24). The amounts reported for the weekly/monthly frequencies were converted to daily equivalents by dividing the total amount consumed during the specified period by the corresponding number of days (25) (see Supplementary Table S2 for details). For instance, if a participant reported consuming fruits 2–3 times per week with a medium-sized portion, which was converted to a standard portion size of 150 g, the conversion was as follows: daily equivalent = amount of food consumed (g) × average reported frequency/number of days. Therefore, in this example, the daily equivalent for fruit consumption was 150 g × 2.5/7 = 53.5 g. Furthermore, these daily food intake values were entered into DietCal software version 15.1.3 (26) using the Indian database to estimate macronutrient intakes (energy, carbohydrate, protein, and fat) and micronutrient intakes (iron, vitamin B12, calcium, and zinc).

### Statistical analysis

The raw data were entered and cleaned for extreme values and ambiguous data in MS Excel. The responses for all categorical variables were numerically coded and exported to the Statistical Packages for Social Sciences (SPSS) software version 30.0 for data analysis (27). The Shapiro–Wilk test indicated a non-normal distribution, supporting the use of non-parametric tests. The continuous variables, such as age, height, weight, and BMI, were expressed as mean ± standard deviation (SD). The categorical sociodemographic variables, including sex, country of origin, marital status, standard of living, education, sponsorship support, current living arrangement, length of stay in India, and ability to speak Hindi for food purchase, were reported as frequencies and percentages. Similarly, changes in eating behaviors before and after migration and the perceived factors influencing food acculturation were summarized using frequencies and percentages. Inferential statistics were performed using non-parametric tests, including multinomial and binomial logistic regression and the Wilcoxon signed-rank test. To investigate the association between sociodemographic determinants and eating behaviors post-migration, multinomial or binomial logistic regression models were fitted, with a separate model for each eating behavior as the dependent variable and all sociodemographic variables as categorical predictors. Furthermore, the Wilcoxon signed-rank test was used to compare food consumption frequencies before and after migration, as well as differences in nutrient intake before and after migration by region of origin. This region-of-origin-specific subgroup and overall sample analysis were conducted to explore whether nutrient intakes differed among IS from different regions of origin. The level of significance was set at *p* ≤ 0.05.

## Results

### Sociodemographic profile

A total of 320 IS participated in the study, with a mean age of 22.5 ± 4.1 years, height of 169.7 ± 10.3 cm, pre-migration weight of 65.2 ± 14.3 kg, post-migration weight of 67.0 ± 14.1 kg, pre-migration BMI of 22.58 ± 4.1 kg/m^2^, and post-migration BMI of 23.18 ± 3.9 kg/m^2^ (Supplementary Table S1). More than half (63.3%, *n* = 207) of the participants were men and unmarried (95%, *n* = 303), had a “medium” standard of living (86.2%, *n* = 276), and were enrolled in an “undergraduate degree” program (62.1%, *n* = 203). The length of stay for most participants (63.8%, *n* = 204) was 7–12 months in Pune, India. By region of origin, the study included more African students (34.7%, *n* = 114) than South Asian students (34%, *n* = 112). For more than half of the students (55.3%, *n* = 177), education was sponsored by their parents/guardians, whereas 35% (*n* = 113) received scholarships. While the majority lived in hostels/dormitories (57%, *n* = 182), 37% (*n* = 118) lived in rented apartments. More than half of the students(54.1%, *n* = 173) reported being able to speak Hindi to facilitate food purchases (Table 1).

**Table 1:** Sociodemographic information of study participants.

Continuous variables		Mean ± SD
Age (years)		22.5 **±** 4.11
Height (cm)		169.7 **±** 10.3
Weight (kg)	Pre-migration	65.2 **±** 14.3
	Post-migration	67.0 **±** 14.3
Body mass index (kg/m^2^)	Pre-migration	22.58 **±** 4.1
	Post-migration	23.18 **±** 3.9

Categorical variables		*N* (%)
Sex	Male	207 (63.3)
	Female	113 (35)
Marital Status	Single	303 (95)
	Married	15 (5)
	Divorced	2 (0.6)
Standard of living	Low	18 (5.6)
	Medium	276 (86.2)
	High	26 (8.1)
Education	Diploma/certificate course	31 (10)
	Undergraduate degree	203 (63.4)
	Postgraduate degree	86 (27)
Length of stay in Pune	3 to ≤ 6 months	27 (8.4)
	7 to 12 months	204 (63.8)
	13 to 24 months	89 (27.8)
Region of origin	Africa	114 (34.7)
	South Asia	112 (34)
	Central Asia	15 (4.6)
	Middle East	74 (22.5)
	Western	5 (1.5)
Sponsorship	Parents/Guardians	177 (55.3)
	Self-sponsored	30 (9.4)
	Scholarship	113 (35.3)
Current living arrangement	College hostel/dormitory	182 (57)
	Paying guest/with relative/family	20 (6.2)
	Rented apartment	118 (37)
Hindi-speaking ability for food purchase	Yes	147 (46)
	No	173 (54.1)

### Perceived factors influencing food acculturation patterns among international students

Table 2 summarizes the perceived factors influencing food acculturation patterns among international students. Most participants agreed or strongly agreed that previous migration experience (29.1%), orientation to Indian food culture (28.1%), and similarities in food (31.2%) facilitated their acculturation process. Regarding dietary changes, about half of the participants reported retaining their non-vegetarian dietary pattern, strongly disagreeing (48.1%) with switching to vegetarianism, while many agreed (40%) that their non-vegetarian food intake had decreased post-migration. Most participants (43.1%) agreed that they consumed sufficient Indian food to satisfy their hunger; however, 18.1% were also “not sure.” Increased fruit and vegetable intake was reported, with the majority agreeing (32%) and strongly agreeing (47%). The perceived food insecurity explored in this section was based on participants’ reported observations across the subdomains of food availability, accessibility, food environment, and food affordability. A greater proportion of participants agreed or strongly agreed that there was sufficient availability of food (33% and 50%, respectively), nutritionally balanced meals in the mess (32.2% and 49%, respectively), good-quality meals (21.2% and 36%, respectively), healthy food options (28.1% and 47.2%, respectively), fresh fruits and vegetables (23.1% and 38.4%, respectively), and convenience/ready-to-eat foods (21.2% and 34.1%, respectively). Food accessibility and the food environment also shape food acculturation patterns, with the majority agreeing or strongly agreeing that they had easy access to balanced meals (45%) and convenience/ready-to-eat foods (51.2%) and maintained a stock of fresh fruits and nuts at their place of residence (28.1%). However, most (32%) also disagreed that they maintained a stock of convenience/ready-to-eat foods. Furthermore, a higher proportion of participants strongly agreed that they had financial support to purchase food (28.4%), including healthy foods (e.g., fruits and nuts) (29.4%) and unhealthy (convenience/ready-to-eat) foods (25%), suggesting greater food affordability. Physical health post-migration was also perceived to have changed, with most participants strongly agreeing that they had experienced hospitalization due to food intolerance (33.4%), gastrointestinal problems (34%), unintentional weight loss (26%), and weight gain (26.2%).

**Table 2:** Perceived factors influencing food acculturation patterns among international students.

Determinants	Strongly agree	Agree	Not sure	Disagree	Strongly disagree
	*N* = (320)				
Previous acculturation experience and present orientation					
Previous migration experience	93 (29.1%)	76(23.8%)	15 (5%)	57 (18%)	79 (25%)
Orientation to the Indian food culture before migration	68 (21.2%)	90 (28.1%)	28 (8.8%)	76 (24%)	58 (18.1%)
Easy adoption due to food similarities	51 (16%)	100 (31.2%)	64 (20%)	62 (19.4%)	43 (13.4%)
Dietary changes post-migration					
Became vegetarian	20 (6.2%)	36 (11.2%)	23 (7.2%)	87 (27.2%)	154 (48.1%)
Enough Indian food consumption to satiate hunger	48 (15%)	138 (43.1%)	58 (18.1%)	49 (15.3%)	27 (8.4%)
Increased fruit and vegetable intake	101 (32%)	149 (47%)	31 (10%)	30 (9.4%)	9 (3%)
Reduced non-vegetarian food intake	57 (18%)	128 (40%)	63 (20%)	56 (17.5%)	16 (5%)
Food availability					
Enough food availability	105 (33%)	159 (50%)	40 (12.5%)	13 (4.1%)	3 (0.9%)
Nutritionally balanced meals availability at mess	103 (32.2%)	156 (49%)	33 (10.3%)	25 (8%)	3 (0.9%)
Availability of good-quality meals	68 (21.2%)	115 (36%)	69 (22%)	55 (17.2%)	13 (4.1%)
Availability of healthy food items (e.g., nuts)	90 (28.1%)	151 (47.2%)	53 (17%)	22 (7%)	4 (1.2%)
Fresh fruits and vegetables	74 (23.1%)	123 (38.4%)	50 (16%)	56 (17.5%)	17 (5.3%)
Convenience/ready-to-eat foods	68 (21.2%)	109 (34.1%)	46 (14.4%)	77 (24.1%)	20 (6.2%)
Food accessibility and environment					
Easy access to balanced meals	82 (26%)	143 (45%)	60 (19%)	32 (10%)	3 (0.9%)
Easy access to convenience/ready-to-eat foods	96 (30%)	164 (51.2%)	41 (13%)	15 (5%)	4 (1.2%)
Stock of convenience/ready-to-eat food at the place of residence	62 (19.4%)	57 (18%)	29 (9.1%)	101 (32%)	71 (22.2%)
Stock of fresh fruits/nuts at the place of residence	63 (20%)	90 (28.1%)	45 (14.1%)	71 (22.2%)	51 (16%)
Food affordability					
Financial support for food availability	63 (20%)	91 (28.4%)	45 (14.1%)	71 (22.2%)	50 (16%)
Affordability to buy nutritious foods like fruits and nuts	49 (15.3%)	94 (29.4%)	62 (19.4%)	66 (21%)	49 (15.3%)
Affordability to buy convenience/ready-to-eat foods	45 (14.1%)	79 (25%)	66 (21%)	77 (24.1%)	53 (17%)
Physical health (post-migration)					
Hospitalization due to food intolerance	62 (19.4%)	107 (33.4%)	72 (22.5%)	48 (15%)	31 (10%)
Faced gastrointestinal problems	62 (19.4%)	108 (34%)	72 (22.5%)	48 (15%)	30 (9.4%)
Unintentional weight loss	57 (18%)	83 (26%)	64 (20%)	73 (23%)	43 (13.4%)
Weight gain	40 (12.5%)	84 (26.2%)	77 (24.1%)	80 (25%)	39 (12.2%)
Sociocultural factors					
Adopted the practice of eating with hands	82 (26%)	44 (14%)	78 (24.4%)	42 (13.1%)	74 (23.1%)
Frequently participate in Indian festivals	48 (15%)	63 (20%)	117 (37%)	47 (15%)	45 (14.1%)
Food choices influenced by Indian friends	74 (23.1%)	59 (18.4%)	93 (29.1%)	62 (19.4%)	32 (10%)
Participation in native social gatherings than the Indian	53 (17%)	55 (17.2%)	108 (34%)	58 (18.1%)	46 (14.4%)
Invitation to enjoy Indian festivals	113 (35.3%)	50 (16%)	74 (23.1%)	32 (10%)	51 (16%)
Religious factors					
Restrict intake of Indian foods due to my religion	37 (12%)	27 (8.4%)	115 (36%)	73 (23%)	68 (21.2%)
Reduced intake of non-veg foods post-migration due to religious norms in India	143 (45%)	58 (18.1%)	59 (18.4%)	27 (8.4%)	33 (10.3%)
Culinary factors					
Indian food is spicy	67 (21%)	36 (11.2%)	85 (27%)	41 (13%)	91 (28.4%)
Acquired basic cooking skills after migration	42 (13.1%)	41 (13%)	80 (25%)	88 (27.5%)	69 (22%)
Self-cooking increased post-migration	63 (20%)	50 (16%)	80 (25%)	46 (14.4%)	81 (25.3%)
Adopted Indian as well as my native cuisine	56 (17.5%)	46 (14.4%)	106 (33.1%)	66 (21%)	46 (14.4%)
Adopted Indian cooking practices	99 (31%)	73 (23%)	70 (22%)	45 (14.1%)	33 (10.3%)
Use of local Indian ingredients in preparing meals	48 (15%)	44 (14%)	92 (29%)	71 (22.2%)	65 (20.3%)
Ordering of Indian food from restaurants	67 (21%)	64 (20%)	111 (35%)	48 (15%)	30 (9.4%)
Increased exploration of local Indian cuisine	57 (18%)	57 (18%)	103 (32.2%)	69 (22%)	34 (11%)
Constantly try to eat my home country’s food	30 (9.4%)	44 (14%)	88 (27.5%)	73 (23%)	85 (27%)
Frequent ordering from food delivery apps	28 (9%)	55 (17.2%)	139 (43.4%)	63 (20%)	35 (11%)
Frequent ordering of non-veg foods from restaurants	40 (12.5%)	48 (15%)	77 (24.1%)	57 (18%)	98 (31%)
Indian food exploration through social media	81 (25.3%)	58 (18.1%)	110 (34.4%)	43 (13.4%)	28 (9%)
Unhealthy food ordering from restaurants	46 (14.4%)	45 (14.1%)	129 (40.3%)	57 (18%)	43 (13.4%)

Sociocultural, religious, and culinary factors may significantly influence food acculturation patterns; however, IS in this study reported mixed responses. Among the sociocultural factors, the most commonly reported were adopting eating with their hands (26%) and being invited to Indian festivals (35%). Moreover, most participants reported being “not sure” about the influence of sociocultural factors, such as frequent participation in Indian festivals (37%), the influence of Indian peers on food choices (29.1%), and participation in non-Indian social gatherings (34%), on their food acculturation patterns. Similarly, for religious factors, such as personal religious practices that restrict the intake of certain Indian foods (36%) and religious practices in the host country that limit the intake of animal-based foods (45%), many participants reported being “not sure” about their influence. Regarding the culinary factors, IS reported mixed responses, with most disagreeing or strongly disagreeing that Indian food was spicy (28.4%), that they had acquired basic cooking skills (27.5%), and that they had increased the frequency of self-cooking (25.3%). Very few (17.5%) of the participants reported adopting both Indian and native cuisine post-migration. The majority (31%, *n* = 99) agreed that they had adopted Indian cooking practices; however, the use of local Indian ingredients was uncommon, with only 15% reporting their use. Although 21% of participants agreed, the majority (35%) reported not being sure about ordering Indian food from restaurants. Similarly, very few agreed that they explored local Indian cuisine (18%), tried to consume food from their home country (9.4%), or frequently used online food delivery apps, whereas the majority reported not being sure (35%, 32.2%, and 27.5%, respectively). A high proportion of participants strongly disagreed (31%) with frequently ordering animal-based foods from restaurants due to food acculturation. Although 25.3% of participants reported exploring Indian food through social media, most (34.4%) were “not sure” about using this medium to discover Indian food options. Furthermore, only 14.4% of participants agreed that they ordered unhealthy food from restaurants, whereas the majority (40.3%) reported being “not sure” about ordering unhealthy food.

### Pre- and post-migration eating behaviors

**Figure 3:**
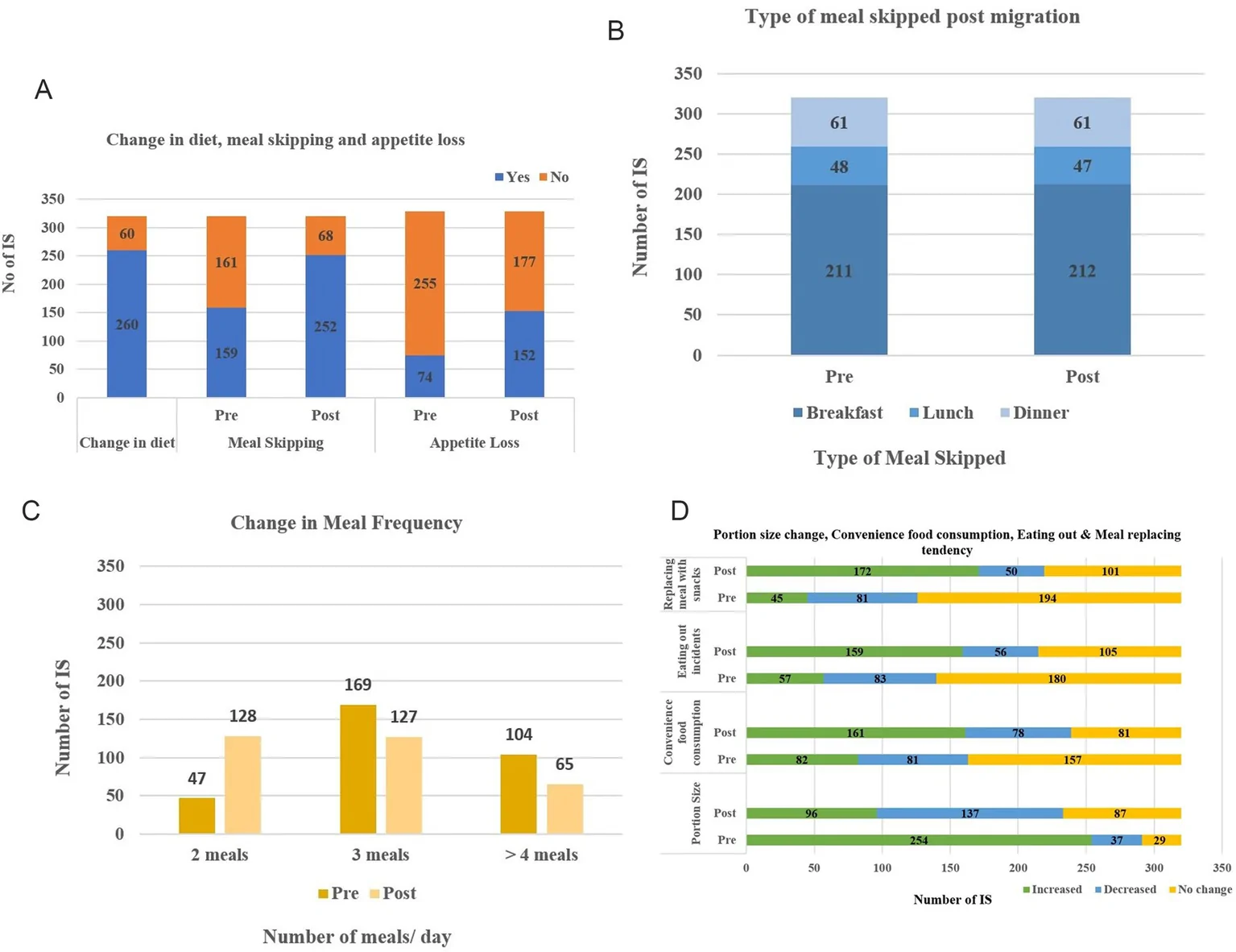
**(A)** Changes in diet, meal skipping, and appetite loss. **(B)** Types of meal skipped post-migration. **(C)** Changes in meal frequency. **(D)** Changes in portion size, convenience food consumption, eating out, and meal-replacement tendency.

IS reported significant changes in their eating behaviors, as depicted in Figures 3A–D. Most participants reported a change in diet (81.2%, n = 260). The highest proportion of participants reported increased meal skipping (post = 79% *vs*. pre = 50%) and appetite loss (post = 47.5% *vs*. pre = 23.1%) post-migration (Figure 3A). Breakfast was reported as the most commonly skipped meal of the day, with no differences between the pre- and post-migration periods (Figure 3B). Regarding meal frequency, a higher proportion of participants reported consuming two meals a day (post = 40% *vs*. pre = 15%). Furthermore, post-migration, fewer participants reported consuming three meals (40%) or more than four meals per day (20.3%) (Figure 3C). Most participants (43%) also reported a decrease in portion size, whereas more than half reported increased consumption of convenience foods (50.3%), eating out (50%), and replacing meals with snacks (54%) (Figure 3D).

### Sociodemographic factors associated with eating behaviors post-migration

Overall, significant associations between sociodemographic factors and post-migration eating behaviors were observed among IS. Young age (18–22 years) was significantly associated with a decrease in portion size [Exp B = 3.19 (CI: 0.97–10.5), *p* = 0.05]. Men were significantly associated with breakfast skipping [Exp B = 1.84 (CI: 1.03–3.28), *p* = 0.04], decreased portion size [Exp B = 0.48 (CI: 0.25–0.93), *p* = 0.03], increased [Exp B = 0.17 (CI: 0.08–0.36), *p* = 0.000] as well as decreased consumption of convenience foods [Exp B = 0.27 (CI: 0.12–0.63), *p* = 0.002], loss of appetite [Exp B = 0.52 (CI: 0.30–0.89), *p* = 0.02], and increased eating out [Exp B = 0.49 (CI: 0.27–0.90), *p* = 0.022]. Marital status was a significant predictor of various unhealthy eating behaviors. “Single” marital status was significantly associated with increased dinner skipping [Exp B = 0.09 (CI: 0.01–0.80), *p* = 0.03]. Furthermore, those belonging to a “low” standard of living group showed increased convenience food consumption [Exp B = 6.99 (CI: 1.03–47.3), *p* = 0.04], higher frequency of eating out [Exp B = 4.65 (CI: 0.95–22.8), *p* = 0.05], whereas increased meal-replacing behavior was observed in both “low” [Exp B = 17.5 (CI: 3.16–97.5), *p* = 0.001] and “medium” [Exp B = 7.93 (CI: 2.35–26.7), *p* = 0.001] standard of living groups. Students enrolled in “Diploma” courses were most likely to consume three meals per day [Exp B = 1.92 (CI: 0.97–3.77), *p* = 0.05] and to report increased meal skipping [Exp B = 0.14 (CI: 0.03–0.58), *p* = 0.007]. A length of stay of 3–6 months [Exp B = 0.26 (CI: 0.07–0.87), *p* = 0.03] and 7–12 months [Exp B = 0.42 (CI: 0.19–0.89), *p* = 0.02] both increased the odds of convenience food consumption. Moreover, those with a shorter duration of stay (3–6 months) showed loss of appetite [Exp B = 0.33 (CI: 0.11–0.96), *p* = 0.04], increased [Exp B = 0.27 (CI: 0.09–0.80), *p* = 0.02], and decreased eating out [Exp B = 0.17 (CI: 0.03–0.87), *p* = 0.034]. By region of origin, those from South Asia [Exp B = 0.41 (CI: 0.19–0.89), *p* = 0.02] and Central Asia tended to skip breakfast [Exp B = 1.43 (CI: 1.21–4.16), *p* = 0.000]. Increased consumption of convenience foods [Exp B = 0.11 (CI: 0.01–0.77), *p* = 0.03] and loss of appetite [Exp B = 0.12 (CI: 0.02–0.66), *p* = 0.01] were observed in Central Asians. Increased eating out was observed among participants from Africa [Exp B = 0.42 (CI: 0.19–0.94), *p* = 0.003], South Asia [Exp B = 0.28 (CI: 0.12–0.71), *p* = 0.007], and Central Asia [Exp B = 0.12 (CI: 0.03–0.52), *p* = 0.005]. Those who self-sponsored [Exp B = 6.05 (CI: 1.29–28.8), *p* = 0.02] their education had three meals per day post-migration. Furthermore, those living as “Paying guest/with relative” had increased odds of meal skipping [Exp B = 0.36 (CI: 0.13–0.99), *p* = 0.04] and decreased consumption of convenience foods [Exp B = 0.09 (CI: 0.01–0.86), *p* = 0.04]. Despite being able to speak local language (Hindi) for food purchasing, participants still had higher odds of appetite loss [Exp B = 0.48 (CI: 0.26–0.89), *p* = 0.02) (Tables 3, 4).

**Table 3:** Sociodemographic factors associated with eating behaviors post-migration.

Variables	Dietary behaviors	Number of meals/days (>4 meals^⸶^, *N* = 65)				Meal skipping (No^⸶^, *N* = 68)		Type of meal skipped (lunch^⸶^, *N* = 47)				Portion size (no change^⸶^, (*N* = 87)			
	Categories	2 meals (*N* = 128)		3 meals (*N* = 128)		Yes (*N* = 252)		Breakfast (*N* = 212)		Dinner (*N* = 61)		Increased (*N* = 96)		Decreased (*N* = 137)	
		Exp B (CI)	*p*-value	Exp B (CI)	*p*-value	Exp B	*p*-value	Exp B (CI)	*p*-value	Exp B (CI)	*p*-value	Exp B (CI)	*p*-value	Exp B (CI)	*p*-value
Age (years)	18–22	1.70 (0.71–4.01)	0.23	0.79 (0.35–1.7)	0.57	1.55 (0.75–3.21)	0.24	0.77 (0.33–1.79)	0.54	0.43 (0.15–1.29)	0.13	2.32 (0.64–8.43)	0.19	3.19 (0.97–10.5)	**0.05**
	23–26	1.51 (0.55–4.20)	0.42	1.11 (0.43–2.8)	0.82	1.14 (0.50–2.61)	0.75	1.20 (0.43–3.37)	0.73	1.51 (0.44–5.18)	0.51	0.99 (0.34–2.94)	0.99	1.95 (0.69–5.48)	0.20
	≥27^⸶^	-													
Sex	Male	1.04 (0.56–1.94)	0.89	1.14 (0.61–2.13)	0.67	0.85 (0.48–1.49)	0.36	1.84 (1.03–3.28)	**0.04**	2.14 (0.96–4.76)	0.06	0.84 (0.41–1.72)	0.63	0.48 (0.25–0.93)	**0.03**
	Female^⸶^	—													
Marital status	Single	2.03 (0.49–8.40)	0.33	0.86 (0.25–2.90)	0.80	1.15 (0.36–3.64)	0.81	0.38 (0.05–3.03)	0.36	0.09 (0.01–0.80)	**0.03**	0.37 (0.09–1.57)	0.18	0.68 (0.15–3.07)	0.62
	Married^⸶^														
Standard of living	Low	0.59 (0.11–3.10)	0.54	1.84 (0.54–6.29)	0.33	1.84 (0.41–8.36)	0.43	1.14 (0.21–6.16)	0.88	0.50 (0.07–3.69)	0.49	4.39 (0.75–25.5)	0.09	2.81 (0.48–16.3)	0.25
	Medium	0.28 (0.06–1.26)	0.09	0.66 (0.23–1.93)	0.45	1.38 (0.55–3.45)	0.48	0.98 (0.31–3.11)	0.98	0.33 (0.09–1.19)	0.09	1.65 (0.55–5.00)	0.37	1.65 (0.56–4.79)	0.36
	High^⸶^	—													
Education	Diploma	1.83 (0.49–6.76)	0.36	1.92 (0.97–3.77)	**0.05**	1.18 (0.45–3.11)	0.74	2.03 (0.53–7.75)	0.29	1.09 (0.21–3.75)	0.91	0.74 (0.17–3.17)	0.68	0.52 (0.12–2.14)	0.36
	UG	2.67 (0.80–8.92)	0.10	1.09 (0.57–2.11)	0.78	1.43 (0.79–2.63)	0.23	0.77 (0.39–1.52)	0.45	0.48 (0.20–1.15)	0.10	0.82 (0.30–2.22)	0.69	1.05 (0.42–2.64)	0.91
	PG degree^⸶^	—													
Length of stay (months)	3 to 6	3.01 (0.59–15.2)	0.18	2.90 (0.59–14.1)	0.18	0.54 (0.21–1.40)	0.20	1.62 (0.48–5.38)	0.43	0.50 (0.08–3.10)	0.46	1.41 (0.36–5.43)	0.62	2.26 (0.64–8.07)	0.21
	7 to 12	1.02 (0.51–2.04)	0.94	0.65 (0.33–1.27)	0.21	1.11 (0.60–2.06)	0.73	1.05 (0.55–2.00)	0.88	0.70 (0.31–1.60)	0.39	1.03 (0.49–2.13)	0.94	1.71 (0.85–3.41)	0.13
	13–24^⸶^	—													
Region of origin	Africa	0.74 (0.32–1.71)	0.48	0.52 (0.22–1.22)	0.13	0.91 (0.45–1.87)	0.79	0.76 (0.33–1.71)	0.50	1.03 (0.34–3.10)	0.96	0.90 (0.36–2.27)	0.82	0.66 (0.28–1.56)	0.35
	South Asia	0.49 (0.27–1.13)	0.09	0.60 (0.26–1.38)	0.23	0.72 (0.35–1.45)	0.36	0.41 (0.19–0.89)	**0.02**	0.55 (0.19–1.63)	0.28	0.65 (0.23–1.85)	0.41	0.39 (0.15–1.05)	0.06
	Central Asia	0.40 (0.10–1.57)	0.19	0.25 (0.06–1.10)	0.06	1.52 (0.31–7.48)	0.60	1.43 (1.21–4.16)	**0.000**	0.30 (0.12–3.11)	0.65	4.55 (0.69–29.7)	0.11	0.89 (0.13–5.99)	0.90
	Middle East^⸶^														
Sponsorship	Parents/guardian	0.75 (0.40–1.41)	0.37	1.04 (0.55–1.99)	0.89	0.79 (0.43–1.44)	0.43	1.24 (0.65–2.34)	0.52	1.64 (0.66–4.08)	0.28	1.20 (0.56–2.57)	0.63	0.61 (0.30–1.22)	0.16
	Self-sponsored	1.77 (0.35–8.98)	0.49	6.05 (1.29–28.8)	**0.02**	0.43 (0.17–1.06)	0.06	4.48 (0.97–20.7)	0.28	3.75 (0.58–24.4)	0.16	0.75 (0.22–2.49)	0.34	0.47 (0.16–1.43)	0.19
	Scholarship^⸶^	—													
Living arrangement	Hostel/dorm	0.80 (0.42–1.51)	0.49	0.77 (0.20–2.90)	0.69	0.95 (0.53–1.69)	0.85	1.12 (0.57–2.20)	0.73	0.59 (0.25–1.40)	0.23	0.95 (0.46–1.97)	0.89	1.24 (0.62–2.48)	0.54
	PG/relative	0.88 (0.46–1.67)	0.70	1.07 (0.29–3.88)	0.91	0.36 (0.13–0.99)	**0.04**	0.60 (0.19–1.83)	0.34	0.14 (0.01–1.33)	0.08	0.35 (0.07–1.65)	0.18	1.12 (0.33–3.79)	0.85
	Apartment^⸶^	—													
Hindi-speaking ability for food purchase	Yes	0.75 (0.41–1.37)	0.34	1.30 (0.71–2.37)	0.39	0.70 (0.41–1.19)	0.19	0.68 (0.38–1.19)	0.18	0.63 (0.29–1.35)	0.23	1.28 (0.59–2.79)	0.53	0.91 (0.44–1.86)	0.79
	No^⸶^	—													

The table presents multinomial/binomial regression analysis. ^⸶^Indicates the reference category. PG: paying guest, UG: undergraduate, PG: postgraduate degree. *p*-values ≤ 0.05 were considered significant as indicated with “bold” values.

**Table 4:** Sociodemographic factors associated with eating behaviors post-migration.

Variables	Dietary behaviors	Convenience food consumption (no change^⸶^, *N* = 81)				Appetite loss (no change^⸶^, *N* = 171)		Eating out incidents (no change^⸶^, *N* = 105)				Meal replacement with snacks (no change^⸶^, *N* = 101)			
	Categories	Increased (*N* = 161)		Decreased (*N* = 78)		Yes (*N* = 149)		Increased (*N* = 159)		Decreased (*N* = 56)		Increased (*N* = 171)		Decreased (*N* = 48)	
		Exp B (CI)	*p*-value	Exp B (CI)	*p*-value	Exp B	*p*-value	Exp B (CI)	*p*-value	Exp B (CI)	*p*-value	Exp B (CI)	*p*-value	Exp B (CI)	*p*-value
Age (years)	18–22	1.74 (0.45–6.67)	0.42	1.04 (0.24–4.62)	0.95	1.26 (0.45–3.520	0.66	1.70 (0.55–5.27)	0.35	1.87 (0.42–8.39)	0.41	1.58 (0.49–4.76)	0.46	2.57 (0.52–12.8)	0.25
	23–26	1.05 (0.34–3.24)	0.93	0.82 (0.23–2.95)	0.76	0.82 (0.33–2.03)	0.67	1.81 (0.66–4.95)	0.24	1.53 (0.43–5.51)	0.51	0.76 (0.29–1.99)	0.58	0.88 (0.22–3.56)	0.85
	≥27^⸶^														
Sex	Male	0.17 (0.08–0.36)	**0.000**	0.27 (0.12–0.63)	**0.002**	0.52 (0.30–0.89)	**0.02**	0.49 (0.27–0.90)	**0.022**	1.06 (0.47–2.39)	0.89	0.58 (0.32–1.07)	0.08	0.87 (0.37–2.04)	0.75
	Female^⸶^														
Marital status	Single	1.67 (0.41–6.88)	0.47	2.31 (0.47–11.3)	0.30	1.14 (0.34–3.81)	0.83	1.51 (0.41–5.59)	0.54	1.51 (0.32–7.22)	**0.60**	1.18 (0.33–4.25)	0.80	1.11 (0.20–6.08)	0.89
	Married^⸶^														
Standard of living	Low	6.99 (1.03–47.3)	**0.04**	4.17 (0.51–34.3)	0.18	1.46 (0.37–5.70)	0.58	4.65 (0.95–22.8)	**0.05**	1.05 (0.12–9.54)	0.96	17.5 (3.16–97.5)	**0.001**	0.19 (0.01–2.78)	0.23
	Medium	1.65 (0.56–4.90)	0.36	1.23 (0.35–4.32)	0.74	1.10 (0.43–2.83)	0.84	1.67 (0.61–4.58)	0.31	1.12 (0.32–3.92)	0.85	7.93 (2.35–26.7)	**0.001**	0.74 (0.24–2.29)	0.59
	High^⸶^														
Education	Diploma	1.14 (0.26–4.89)	0.86	2.04 (0.37–11.1)	0.41	1.41 (0.41–4.86)	0.57	0.36 (0.09–1.47)	0.15	3.05 (0.55–16.9)	0.20	0.14 (0.03–0.58)	**0.007**	1.58 (0.28–8.94)	0.60
	UG	0.95 (0.36–2.50)	0.92	1.35 (0.46–3.96)	0.58	1.65 (0.73–3.69)	0.22	0.65 (0.26–1.56)	0.33	0.40 (0.13–1.28)	0.12	0.88 (0.38–2.06)	0.77	0.60 (0.17–2.12)	0.49
	PG degree^⸶^														
Length of stay (months)	3 to 6	0.26 (0.07–0.87)	**0.03**	0.31 (0.07–1.32)	0.11	0.33 (0.11–0.96)	**0.04**	0.27 (0.09–0.80)	**0.02**	0.17 (0.03–0.87)	**0.034**	0.43 (0.15–1.25)	0.12	0.25 (0.04–1.56)	0.14
	7 to 12	0.42 (0.19–0.89)	**0.02**	0.64 (0.27–1.52)	0.32	1.25 (0.69–2.25)	0.45	0.65 (0.33–1.26)	1.20	0.67 (0.28–1.57)	0.35	1.02 (0.53–1.94)	0.95	1.38 (0.55–3.46)	0.49
	13–24 ⸶														
Region of origin	Africa	0.77 (0.33–1.86)	0.57	2.02 (0.72–5.68)	0.18	0.73 (0.36–1.45)	0.36	0.42 (0.19–0.94)	**0.003**	2.66 (0.81–8.79)	0.10	0.63 (0.28–1.39)	0.26	2.26 (0.69–7.33)	0.17
	South Asia	0.55 (0.21–1.44)	0.22	1.17 (0.36–3.74)	0.79	0.95 (0.43–2.10)	0.91	0.28 (0.12–0.71)	**0.007**	1.07 (0.27–4.27)	0.92	0.55 (0.22–1.35)	0.19	1.26 (0.33–4.78)	0.73
	Central Asia	0.11 (0.01–0.77)	**0.03**	2.12 (0.40–11.1)	0.37	0.12 (0.02–0.66)	**0.01**	0.12 (0.03–0.52)	**0.005**	1.58 (0.24–10.5)	0.63	0.47 (0.11–2.01)	0.31	2.22 (0.32–15.5)	0.42
	Middle East^⸶^														
Sponsorship	Parents/guardian	0.64 (0.31–1.32)	0.23	0.54 (0.24–1.19)	0.13	0.84 (0.47–1.48)	0.54	0.66 (0.35–1.24)	0.19	0.73 (0.32–1.68)	0.46	0.57 (0.29–1.10)	0.09	0.58 (0.24–1.41)	0.23
	Self-sponsored	1.68 (0.52–5.52)	0.39	0.62 (0.14–2.82)	0.54	0.53 (019–1.47)	0.23	1.17 (0.41–3.39)	0.76	0.56 (0.13–2.53)	0.45	1.64 (0.55–4.89)	0.37	0.36 (0.07–1.82)	0.21
	Scholarship^⸶^														
Living arrangement	Hostel/Dorm	0.92 (0.45–1.86)	0.81	1.21 (0.54–2.72)	0.64	1.46 (0.82–2.60)	0.19	0.98 (0.52–1.86)	0.96	1.44 (0.61–3.38)	0.41	0.96 (0.50–1.83)	0.90	1.17 (0.48–2.84)	0.71
	PG/Relative	0.39 (0.12–1.33)	0.13	0.09 (0.01–0.86)	**0.04**	0.96 (0.30–3.01)	0.94	0.90 (0.30–2.67)	0.85	0.36 (0.03–1.33)	0.69	0.66 (0.22–2.02)	0.47	1.14 (0.01–1.41)	0.45
	Apartment^⸶^														
Hindi-speaking ability for food purchase	Yes	0.63 (0.29–1.32)	0.22	0.54 (0.23–1.26)	0.15	0.48 (0.26–0.89)	**0.02**	1.03 (0.53–2.02)	0.92	0.99 (0.41–2.41)	0.98	1.02 (0.52–3.03)	0.94	1.19 (0.48–2.97)	0.70
	No^⸶^	—													

The table presents multinomial/binomial regression analysis. ^⸶^Indicates the reference category. PG: paying guest, UG: undergraduate, PG: postgraduate degree. *p*-values ≤ 0.05 were considered significant as indicated with “bold” values.

### Food consumption frequency pre- and post-migration

The frequency of food consumption across 20 food groups, before and after migration, is reported descriptively in Supplementary Table S2. A statistically significant decline was observed in the consumption of protein-rich food groups from both non-vegetarian and vegetarian sources. Among the non-vegetarian protein sources, reduced consumption was observed for beef (Z = −6.453, *p* < 0.001), mutton (Z = −5.807, *p* < 0.001), fish (Z = −3.297, *p* = 0.001), seafood (Z = −3.466, *p* = 0.001), poultry and eggs (Z = −3.434, *p* = 0.001), milk (Z = −3.877, *p* < 0.001), and milk products (Z = −1.961, *p* = 0.05). Similarly, reduced consumption was observed for vegetarian protein sources such as pulses and legumes (Z = −2.310, *p* = 0.021) and nuts and oilseeds (Z = −2.697, *p* = 0.007). Additionally, the frequency of consuming green leafy vegetables (Z = −2.060, *p* = 0.039) and common fruits such as apples and bananas (Z = −2.589, *p* = 0.010) was significantly reduced post-migration. No statistically significant changes were noted for cereals, group A vegetables (fruit vegetables), group B vegetables (roots and tubers), exotic fruits, fats/oils, sugars, convenience/ready-to-eat foods, and dietary supplements (all *p* > 0.05) (Table 5).

**Table 5:** Comparison between food consumption frequency pre- and post-migration.

Food group	Frequency pre-migration (M ± SD)	Frequency post-migration (M ± SD)	*Z*	*p*-value
Cereal group A (wheat, bread)	2.50 ± 1.48	2.33 ± 1.49	−1.418	0.156
Cereal group B (Rice)	2.30 ± 1.28	2.37 ± 1.07	−0.899	0.368
Beef	6.09 ± 1.47	4.68 ± 1.97	−6.453	0.000*
Mutton	5.85 ± 1.53	4.83 ± 1.80	−5.807	0.000*
Fish	5.80 ± 1.39	5.03 ± 1.79	−3.297	0.001*
Seafood (crabs, oysters)	6.61 ± 1.07	6.30 ± 1.46	−3.466	0.001*
Poultry and eggs	3.76 ± 1.54	3.32 ± 1.43	−3.434	0.001*
Pulses and legumes	3.25 ± 1.69	2.91 ± 1.43	−2.310	0.021*
Nuts and oilseeds	4.57 ± 1.94	4.16 ± 1.83	−2.697	0.007*
Milk	3.58 ± 2.02	3.08 ± 1.95	−3.877	0.000*
Milk product	3.47 ± 1.55	3.20 ± 1.61	−1.961	0.05*
Vegetable group A (fruit vegetables)	3.06 ± 1.60	2.77 ± 1.29	−1.434	0.152
Vegetable group B (roots and tubers)	3.14 ± 1.23	3.14 ± 1.20	−0.053	0.958
Vegetable group C (green leafy vegetables)	3.78 ± 1.61	3.34 ± 1.72	−2.060	0.039*
Fruit (common: apple, banana)	3.08 ± 1.15	2.69 ± 1.20	−2.589	0.010*
Fruit (exotic, kiwi, berries)	5.00 ± 1.87	4.60 ± 1.81	−1.571	0.116
Sugar	2.46 ± 1.47	2.36 ± 1.35	−0.414	0.679
Fat/oils	2.11 ± 1.06	2.12 ± 1.28	−0.379	0.705
Convenience/ready-to-eat foods	3.57 ± 1.63	3.89 ± 1.75	−1.262	0.207
Dietary supplements, including herbal	5.80 ± 1.91	6.24 ± 1.61	−1.766	0.077

Food consumption frequency units are 1 = twice daily, 2 = daily, 3 = 2–3 times per week, 4 = weekly, 5 = twice monthly, 6 = monthly, and 7 = never. The values in bold and marked with ‘*’ indicate statistically significant values with *p* < 0.05.

### Post-migration changes in anthropometric measurements and nutrient intake

Table 6 illustrates the mean changes in anthropometric measurements and nutrient intake among IS from six regions of origin—Africa, South Asia, the Middle East, Southeast Asia, Central Asia, and Western countries—before and after migration. Overall, body weight increased significantly among African (65.9 **±** 12.6 *vs*. 63.8 ± 12.6; Z = −3.63, *p* < 0.001), South Asian (67.2 ± 12.0 *vs*. 64.7 ± 13.8; Z = −3.04, *p* = 0.002), and Middle Eastern students (71.1 ± 13.7 *vs*. 69.1 ± 16.0; Z = −3.05, *p* = 0.002). Similarly, BMI increased significantly after migration among students from Africa (22.2 ± 3.13 *vs*. 21.6 ± 3.16; Z = −4.28, *p* < 0.001), South Asia (25.6 ± 2.91 *vs*. 25.3 ± 3.08; Z = −2.58, *p* = 0.010), and the Middle East (26.3 ± 3.74 *vs*. 24.5 ± 5.20; Z = −2.16, *p* = 0.03). Energy intake increased among students from Africa (2013.2 ± 901.0 *vs*. 1820.4 ± 575.4; Z = −2.22, *p* = 0.026), South Asia (1949.8 ± 666.3 *vs*. 1788.9 ± 496.5; Z = −1.96, *p* = 0.05), the Middle East (2040.9 ± 694.5 *vs*. 1713.2 ± 497.2; Z = −3.62, *p* < 0.001), and Western countries (1,320 ± 141.7 *vs*. 1088.2 ± 280.0; Z = −2.03, *p* = 0.04). Moreover, carbohydrate intake increased only among students from Africa (255.7 ± 114.0 *vs*. 237.4 ± 93.9; Z = −2.89, *p* = 0.004) and Western countries (270.3 ± 118.7 *vs*. 231.0 ± 93.6; Z = −2.99, *p* = 0.003). Conversely, protein intake decreased significantly among students from all regions except Western countries, including Africa (91.0 ± 37.0 *vs*. 76.0 ± 24.9; Z = −4.39, *p* < 0.001), South Asia (89.5 ± 33.2 *vs*. 72.6 ± 25.9; Z = −5.63, *p* < 0.001), Central Asia (93.0 ± 35.7 *vs*. 68.3 ± 30.1; Z = −3.23, *p* = 0.001), and the Middle East (92.6 ± 33.5 *vs*. 67.5 ± 19.4; Z = −5.54, p = 0.001). While overall fat intake increased significantly post-migration (64.3 ± 32.2 *vs*. 59.0 ± 27.8; Z = −2.48, *p* = 0.01), this increase was observed only among students from the Middle East (67.0 ± 32.1 *vs*. 56.3 ± 23.4; Z = −2.67, *p* = 0.008).

**Table 6:** Mean changes in anthropometric measurements and nutrient intake before and after migration by region of origin.

Variable	Africa (*N* = 114)		South Asia (*N* = 112)		Central Asia (*N* = 15)		Middle East (*N* = 74)		Western (*N* = 5)		Overall (*N* = 320)	
	Before	After	Before	After	Before	After	Before	After	Before	After	Before	After
	Mean ± SD		Mean ± SD		Mean ± SD		Mean ± SD		Mean ± SD		Mean ± SD	
Weight (kg) Z (*p*-value)	63.8 ± 12.1	65.9 **±** 12.6	64.7 ± 13.8	67.2 ± 12.0	62.2 ± 11.6	61.5 ± 8.8	69.1 ± 16.0	71.1 ± 13.7	65.5 ± 13.9	66.1 ± 13.6	65.3 ± 13.7	67.4 ± 12.7
	−3.63 (0.000)*		−3.04 (0.002)*		−0.67 (0.49)		−3.05 (0.002)*		−0.36 (0.71)		−5.61 (0.000)*	
BMI (kg/m^2^) Z (*p*-value)	21.6 ± 3.16	22.2 ± 3.13	22.5 ± 3.97	23.07 ± 3.74	22.4 ± 5.33	23.4 ± 2.52	23.8 ± 4.96	24.5 ± 5.18	25.0 ± 3.72	25.6 ± 4.48	22.58 ± 4.1	23.18 ± 3.9
	−4.28 (0.000)*		−2.66 (0.008)*		**−**0.38 (0.70)		−2.27 (0.023)*		−1.28 (0.197)		−5.31 (0.000)*	
Energy (kcal) Z (*p*-value)	1820.4 ± 575.4	2013.2 ± 901.0	1788.9 ± 496.5	1949.8 ± 666.3	1801.8 ± 444.8	1846.9 ± 492.8	1713.2 ± 497.2	2040.9 ± 694.5	1088.2 ± 280.0	1,320 ± 141.7	1781.4 ± 520.1	1928.4 ± 755
	−2.22 (0.026)*		−1.96 (0.05)*		−0.22 (0.82)		−3.62 (0.000)*		−2.03 (0.04)*		−4.13 (0.000)*	
CHO (g) Z (*p*-value)	237.4 ± 93.9	255.7 ± 114.0	249.3 ± 104.5	238.2 ± 101.3	260.8 ± 122.5	243.5 ± 87.7	215.2 ± 88.5	218.3 ± 87.7	231.0 ± 93.6	270.3 ± 118.7	188.8 ± 89.2	232.2 ± 69.4
	−2.89 (0.004)*		−1.32 (0.18)		−1.24 (0.21)		−0.23 (0.82)		−2.99 (0.003)*		−1.21 (0.22)	
Protein (g) Z (*p*-value)	91.0 ± 37.0	76.0 ± 24.9	89.5 ± 33.2	72.6 ± 25.9	93.0 ± 35.7	68.3 ± 30.1	92.6 ± 33.5	67.5 ± 19.4	73.2 ± 30.7	78.0 ± 18.4	90.7 ± 34.6	72.5 ± 24.4
	−4.39 (0.000)*		−5.63 (0.000)*		−3.23 (0.001)*		−5.54 (0.000)*		−0.54 (0.58)		−9.26 (0.000)*	
Fat (g) Z (*p*-value)	64.9 ± 36.7	60.5 ± 30.1	62.4 ± 26.8	57.9 ± 25.0	55.8 ± 22.8	58.8 ± 38.0	56.3 ± 23.4	67.0 ± 32.1	76.6 ± 60.9	94.5 ± 43.9	59.0 ± 27.8	64.3 ± 32.2
	−0.79 (0.42)		−1.54 (0.12)		−0.22 (0.82)		−2.67 (0.008)*		−1.48 (0.13)		−2.48 (0.01)*	
Iron (mg) Z (*p*-value)	18.2 ± 9.8	14.2 ± 7.8	18.6 ± 8.6	13.6 ± 7.9	15.4 ± 8.4	10.2 ± 6.1	17.7 ± 8.4	13.3 ± 8.3	15.6 ± 10.9	17.3 ± 5.6	18.0 ± 9.0	13.6 ± 7.9
	−3.84 (0.000)*		−2.50 (0.012)*		−4.00 (0.000)*		−0.67 (0.50)		−0.67 (0.50)		−7.92 (0.000)*	
B12 (μg) Z (*p*-value)	6.2 ± 4.2	4.5 ± 2.5	5.7 ± 4.1	4.4 ± 2.8	5.1 ± 2.6	3.4 ± 2.2	5.6 ± 2.8	3.6 ± 1.7	5.5 ± 4.5	5.6 ± 3.2	5.8 ± 3.8	4.2 ± 2.5
	−4.42 (0.000)*		−3.78 (0.000)*		−2.58 (0.010)*		−5.57 (0.000)*		−0.67 (0.50)		−7.99 (0.000)*	
Calcium (mg) Z (*p*-value)	665.3 ± 263.8	622.9 ± 298.8	760.7 ± 314.7	629.4 ± 351.6	805.8 ± 470.7	728.2 ± 410.6	790.1 ± 315.7	605.2 ± 278.7	626.7 ± 207.9	750.9 ± 146.3	734.1 ± 308.9	628.1 ± 318.0
	**−1.70 (0.08)**		−4.35 (0.000)*		−0.51 (0.60)		−4.45 (0.000)*		−1.21 (0.22)		−5.82 (0.000)*	
Zinc (mg) Z (*p*-value)	10.2 ± 4.0	9.2 ± 4.3	10.7 ± 4.3	8.2 ± 4.0	10.5 ± 5.3	6.28 ± 4.3	11.0 ± 4.0	8.0 ± 4.0	7.6 ± 2.7	7.8 ± 3.8	10.6 ± 4.1	8.4 ± 4.2
	−2.35 (0.019)*		−5.31 (0.000)*		−2.49 (0.012)*		−5.31 (0.000)*		−0.36 (0.71)		−7.89 (0.000)*	

*p-value <0.05 = statistically significant; CHO: carbohydrate. The values in bold and marked with ‘*’ indicate statistically significant values with *p* < 0.05.

For micronutrients, intakes of iron (13.6 ± 7.9 *vs*. 18.0 ± 9.0; Z = −7.92, *p* < 0.001), vitamin B12 (4.2 ± 2.5 *vs*. 5.8 ± 3.8; Z = −7.99, *p* < 0.001), calcium (628.1 ± 318.0 *vs*. 734.1 ± 308.9; Z = −5.82, *p* < 0.001), and zinc (8.4 ± 4.2 *vs*. 10.6 ± 4.1; Z = −7.89, *p* < 0.001) decreased significantly among all IS. Iron intake decreased significantly among IS from Africa (14.2 ± 7.8 *vs*. 18.2 ± 9.8; Z = −3.84, *p* < 0.001), South Asia (13.6 ± 7.9 *vs*. 18.6 ± 8.6; Z = −2.50, *p* = 0.012), and Central Asia (10.2 ± 6.1 *vs*. 15.4 ± 8.4; Z = −4.00, *p* < 0.001). Furthermore, vitamin B12 intake decreased among students from Africa (4.5 ± 2.5 *vs*. 6.2 ± 4.2; Z = −4.42, *p* < 0.001), South Asia (4.4 ± 2.8 *vs*. 5.7 ± 4.1; Z = −3.78, *p* < 0.001), Central Asia (3.4 ± 2.2 *vs*. 5.1 ± 2.6; Z = −2.58, *p* = 0.010), and the Middle East (3.6 ± 1.7 *vs*. 5.6 ± 2.8; Z = −5.57, *p* < 0.001). Moreover, calcium intake decreased among students from Africa (622.9 ± 298.8 vs. 665.3 ± 263.8; Z = −1.70, *p* = 0.08), South Asia (629.4 ± 351.6 *vs*. 760.7 ± 314.7; Z = −4.35, *p* < 0.001), and the Middle East (605.2 ± 278.7 *vs*. 790.1 ± 315.7; Z = −4.45, *p* < 0.001). Similarly, zinc intake decreased among IS from Africa (9.2 ± 4.3 *vs*. 10.2 ± 4.0; Z = −2.35, *p* = 0.019), South Asia (8.2 ± 4.0 *vs*. 10.7 ± 4.3; Z = −5.31, *p* < 0.001), Central Asia (6.28 ± 4.3 *vs*. 10.5 ± 5.3; Z = −2.49, *p* = 0.012), and the Middle East (8.0 ± 4.0 *vs*. 11.0 ± 4.0; Z = −5.31, *p* < 0.001).

## Discussion

This study investigated food acculturation patterns, eating behaviors, perceived influencing factors, and differences in anthropometry and nutrient intake by region of origin before and after migration among IS residing in Pune, India. The study highlights four key findings. First, a higher proportion of IS reported that previous migration experience, food availability, accessibility, food environment, affordability, sociocultural factors, religious factors, and culinary factors influenced their food acculturation patterns. Second, a higher proportion of participants reported changes in eating behaviors after migration, which were also associated with sociodemographic factors such as age, sex, marital status, standard of living, education, region of origin, sponsorship, living arrangement, and the ability to speak the local language (Hindi) for food purchasing. Third, a marked decline in the consumption of protein-rich foods from both animal and vegetarian sources, as well as green leafy vegetables and fruits, was observed among IS. Finally, significant changes in anthropometry and nutrient intake were observed across the study population, with differences by region of origin.

Food acculturation patterns were notable among IS in India, with the majority reporting retention of their home-country dietary pattern, which was predominantly non-vegetarian (48.1%, *n* = 154). This finding is consistent with Satia Abouta’s model of dietary acculturation (4), which explains the retention of home-country dietary habits and practices given the strong link between diet and culture. Furthermore, IS also reported sufficient consumption of Indian foods, implying partial adoption of the host country’s dietary patterns, as reported previously (20, 28). This need to maintain “Cultural identity” by adhering to foods from their home country, even after migration, may be explained by the emotional comfort that food provides, physical sustenance, familiarity of taste, and the perceived healthfulness of the native diet (21). In addition to these food acculturation patterns, a reduced intake of non-vegetarian foods, which formed an important component of many participants’ home-country diets, was observed, underscoring the influence of the host country’s food environment and religious food norms (29). India has a deeply rooted food culture strongly influenced by Hindu traditions, in which meat consumption is often limited and avoided on many auspicious days and festivals, contributing to the country’s large vegetarian population (30). Given the widespread practice of vegetarianism in India, the social and institutional food environment predominantly supports vegetarian food choices (31). Although globalization has shifted dietary patterns in India, with easier access to non-vegetarian foods (32), the food consumption of IS in our study may also have been influenced by other factors, such as food affordability, differences in culinary practices, and taste preferences; however, this requires further exploration.

Food insecurity among IS has been noted as a serious health concern in previous studies (22), as the availability, accessibility, and affordability of food in the host country may significantly affect food intake. Contrary to previous studies (6, 22), IS in India reported sufficient availability of nutritionally balanced, high-quality meals, easy access to food, and financial support for both healthy (fruits, vegetables, and nuts) and unhealthy, convenience/ready-to-eat foods. This contradictory finding essentially highlights two important points: first, food availability, accessibility, and affordability of healthy and unhealthy foods may not be the primary perceived factors influencing the food intake and food insecurity status of IS in the Indian context. Second, there is a dire need for qualitative inquiry into these factors and for assessing not only food insecurity but also nutritional insecurity. However, the quantitative assessment of perceived sociocultural, religious, and culinary factors reported by IS in this study partially helps explain the barriers to food acculturation in the Indian context. For instance, the majority of participants were unsure about their participation in Indian festivals and non-Indian social gatherings and the influence of Indian peers as facilitators of food acculturation. These perceptions may lead to limited participation and reduced exposure to the host country’s food culture and festivals. Contrary to these findings, previous studies reported increased participation (33, 34). Despite being invited to celebrate local Indian festivals or to participate in cultural celebrations specifically planned for IS, participants’ participation may still be low because of factors such as time constraints, a lack of peers to accompany them, or disinterest in the planned activities (35), supporting the findings of this study. Among the culinary factors, the acquisition of basic cooking skills, increased self-cooking, lower adoption of Indian cuisine, and limited use of local ingredients were commonly perceived as barriers to food acculturation, corroborating the findings from other studies among IS (6, 11, 36, 37). Furthermore, a high proportion of participants reported adopting Indian cooking practices, which may facilitate food acculturation, consistent with previous studies that reported greater adoption of local cooking practices (6, 11, 22).

Investigation of eating behaviors before and after migration, measured by the number of participants reporting changes over time, provided critical insights into which specific behaviors were altered among IS in India. Overall, the majority of IS reported changes in their eating behaviors, most of which were concerning. An increased tendency to skip meals was observed among IS in this study, consistent with previous findings (38, 39), with breakfast being the most commonly skipped meal. Many IS also reported loss of appetite, corroborating findings from another Indian study (10). This loss of appetite also manifested as a decrease in meal frequency, with participants reporting consuming only two meals per day post-migration (10, 40). Additionally, IS reported significant reductions in portion sizes post-migration, consistent with previous studies (10). Contrary to our findings, a US study reported increased portion sizes, likely due to larger portions served and all-you-can-eat buffet services (11). Moreover, the reduced meal frequency observed in this study (<2 meals/day) contrasts with findings from a study in South Korea, which reported a 14.1% increase in meal frequency among Chinese IS. The reduction in portion size and meal frequency observed in our study may be due to poor adaptation to local Indian cuisine, unfamiliarity with it, limited food options, individual culinary preferences, structural and environmental barriers, or concerns about food hygiene, all of which were reported by a high proportion of participants. These factors may have contributed to reduced caloric intake and altered eating patterns, warranting further investigation. In addition to these perceived influences, reduced portion size and meal skipping may also be attributed to time constraints, academic pressures, food access, and religious and culinary factors, as reported in previous studies (10, 11, 22, 41). Additionally, the consumption of convenience foods, eating out, and replacing meals with snacks increased post-migration, as observed in previous studies (10, 11). Although these eating behaviors are common among young adults, they become even more critical among IS because of their unique context and require further investigation.

Eating behaviors post-migration may also differ based on the sociodemographic profile of IS, as examined in this study. Sociodemographic characteristics, such as age, sex, marital status, standard of living, education, region of origin, sponsorship, living arrangement, and the ability to speak the local language (Hindi) for food purchasing, were associated with different eating behaviors post-migration. Young adults, in particular, reported consuming smaller portions, whereas those living in Western countries have reported the opposite. For example, IS studying in the United States reported that “large portion sizes” were served on campuses (6, 11, 42). Despite having the choice to consume appropriate portion sizes, young adults tend to overeat when large portions are served (43). Whether this finding is specific to Asian countries or is unique to the Indian context requires further investigation. By sex, men were more likely to skip breakfast, report a reduced portion size, increased consumption of convenience foods, loss of appetite, and more frequent eating out, findings that have been consistently observed previously (44–46). This could be attributed to higher self-perceived food literacy (43) or greater body-image and weight concerns among women than among men (47, 48). Although being single was significantly associated with increased dinner skipping, no significant changes in other eating behaviors were observed, perhaps highlighting the unique “international” status rather than marital status. Similar findings were observed in a Korean study, wherein married migrant women showed no significant changes in unhealthy eating habits (49). Similarly, regardless of whether they had a low or medium standard of living, IS showed increased consumption of convenience foods, eating out, and meal-replacing behavior (50). Importantly, recent migration and a shorter length of stay were associated with increased odds of convenience food consumption, loss of appetite, and changes in eating out (both increased and decreased), as also observed in another study from India (10). These findings suggest that individuals may still be in the exploration/adaptation phase of acculturation, which may contribute to the adoption of unhealthy eating behaviors during food acculturation. Additionally, the risk of adopting unhealthy eating behaviors during the early stages of migration warrants further investigation to identify the facilitators and barriers that may influence food acculturation. Future studies may consider the early period after migration as a “window of opportunity” for planning appropriate nutrition education interventions. By region of origin, African, South Asian, Central Asian, and Middle Eastern populations reported unhealthy eating behaviors post-migration, as also observed previously (10, 39). The ability to communicate in the host country’s local language facilitates acculturation, including food acculturation, as previous studies have shown (18, 29). Despite this, IS in our study who spoke Hindi for food purchases still reported undesirable changes in eating behaviors, including loss of appetite. Nevertheless, this finding underscores the need for further investigation, as language barriers may impede healthy eating. Additionally, a lack of familiarity with local terms for vegetables, fruits, and other healthy foods may also affect food intake (51).

A significant decline in the consumption of animal-based foods, particularly beef, mutton, fish, seafood, poultry, and eggs, was observed post-migration, supporting findings from a previous study, which reported similar trends (10). This may be attributed to the religious norms in India, which limit the intake of animal-based foods, as reported descriptively by participants. Furthermore, other reasons may also be practical constraints, such as limited availability, higher cost, lack of storage facilities, and food safety concerns, rather than cultural or religious factors as reported previously (36). Additionally, the consumption of plant-based protein sources, such as lentils and pulses, was observed among IS, but at low frequency. This may reflect partial but poor adaptation to the local food environment of India, despite the higher production and availability of pulses than in other regions of the world (52). This dietary shift was associated with a notable reduction in the intake of essential nutrients, including protein, iron, vitamin B12, calcium, and zinc (53). These findings are consistent with earlier studies reporting micronutrient deficiencies among IS following food acculturation (6, 10). The risk of vitamin B12 deficiency is particularly concerning in predominantly vegetarian settings (54). Additionally, reduced consumption of dairy products may have contributed to lower calcium and zinc intake, further emphasizing the nutritional implications of dietary transitions in this population. Such an unbalanced dietary transition may be associated with low muscle mass and an increased risk of obesity among IS (55), as observed in this study, in which body weight increased slightly without substantial changes in BMI, while IS consistently reported unhealthy eating behaviors. This finding underscores the need for longitudinal research to confirm the long-term effect on the physical health of IS. Overall, the increased energy intake observed in this study may be associated with significantly higher intakes of carbohydrates and fat. In summary, IS in India experienced food acculturation, with undesirable changes in eating behaviors influenced by sociodemographic factors. The majority of participants reported various perceived factors influencing food acculturation patterns. Although participants’ BMI remained within normal limits, their body weight increased slightly. The nutrient intakes differed significantly post-migration among IS. These findings suggest that although IS migrate temporarily to host countries, food acculturation and dietary displacement may significantly affect their short- and long-term physical health.

This study is strengthened by its unique cross-cultural context and its focus on an underexplored and vulnerable population. The food acculturation patterns and post-migration changes in eating behaviors of IS from India observed in this study differ from those in Western countries, making the study setting valuable. The study findings are supported by an overall adequate sample size, and the study questionnaire was comprehensive, validated, and pilot-tested with good reliability. The questionnaire covered critical domains such as food availability, affordability, accessibility, social, cultural, religious, and culinary factors, and eating behaviors, along with a FFQ comprising 20 food groups. Region-of-origin-specific changes in nutrient intake and anthropometry provided a broader understanding of nutritional status. Despite these strengths, the study’s findings should be interpreted cautiously. The exploratory nature of the study prevents the establishment of causal relationships between the variables. Except for the anthropometric data, most data were self-reported, which may have introduced recall and social-desirability bias, especially for retrospective dietary data. Despite following the standardized protocol for participant recruitment, the purposive sampling method limits the generalizability of the findings. Although the overall analysis of the whole population showed remarkable changes in anthropometry and nutrient intake, the smaller subgroup analyses based on region of origin should be interpreted with caution, requiring further investigation. Additionally, anthropometric measurements could be compared across BMI categories in future studies. Dietary/nutritional adequacy may be better assessed in future studies by comparison with the standard recommended dietary allowances (RDAs) and estimated average requirements (EARs), which were not explored in this study due to an ethnically diverse population and differences in standards across regions of origin. Further analysis of other important micronutrients, such as sodium, potassium, vitamin E, vitamin C, and vitamin D, is needed to achieve a comprehensive understanding of dietary/nutritional adequacy. Future research should also examine the long-term effects of unhealthy food acculturation and use qualitative inquiry to better understand other factors and perceptions regarding food acculturation among IS and institutional stakeholders. Finally, this study highlights the significance of planning targeted nutrition interventions and education.

## Conclusion

Food acculturation patterns and eating behaviors among IS differed post-migration, with a higher proportion of participants reporting the influence of various factors, including sociodemographic, cultural, religious, and culinary factors, among IS in Pune, India. Acculturation-related dietary displacements were particularly concerning, as they were associated with reduced consumption of protein-rich foods, green leafy vegetables, and fruits. In addition, sociodemographic factors such as age, sex, marital status, standard of living, education, region of origin, living arrangements, and the ability to speak the local language for food purchasing substantially shaped eating behaviors. Collectively, these findings demonstrate that food acculturation is a complex process that may lead to unintended nutritional consequences affecting the overall health of IS. The study further emphasizes the need for longitudinal and qualitative research to better understand the long-term physical health impacts of food acculturation among IS in the Indian context.

## Data Availability

The raw data supporting the conclusions of this article will be made available by the authors, without undue reservation.
