# Supplementary material for: Food acculturation and nutrient intake among international students in India: an exploratory cross-cultural study
Source: Front Nutr. 2026 Aug 7;13:1865569. doi: 10.3389/fnut.2026.1865569 (PMC13495694; doi:10.3389/fnut.2026.1865569)
Supplement: Supplementary file 1 [file Data_Sheet_1.PDF]

Table 1: Pre- and Post-migration Food Frequency pattern of international students

Food Group		Twice Daily	Daily	2–3 Times/Week	Weekly	Twice Monthly	Monthly	Never
Cereal group A (wheat, bread)	Pre	80 (25.6%)	129 (41.3%)	50 (16.0%)	28 (9.0%)	3 (1.0%)	5 (1.6%)	17 (5.4%)
	Post	56 (17.9%)	130 (41.5%)	64 (20.4%)	37 (11.8%)	5 (1.6%)	6 (1.9%)	15 (4.8%)
Cereal group B (Rice)	Pre	36 (11.4%)	166 (52.7%)	71 (22.5%)	21 (6.7%)	7 (2.2%)	5 (1.6%)	9 (2.9%)
	Post	56 (17.9%)	162 (51.9%)	48 (15.4%)	28 (9.0%)	6 (1.9%)	4 (1.3%)	8 (2.6%)
Beef	Pre	12 (3.7%)	44 (13.5%)	71 (21.7%)	61 (18.7%)	28 (8.6%)	32 (9.8%)	79 (24.2%)
	Post	4 (1.3%)	18 (5.8%)	30 (9.6%)	33 (10.5%)	14 (4.5%)	36 (11.5%)	178 (56.9%)
Mutton	Pre	13 (4.2%)	15 (4.8%)	84 (27.1%)	41 (13.2%)	34 (11.0%)	66 (21.3%)	57 (18.4%)
	Post	4 (1.3%)	8 (2.6%)	52 (16.6%)	56 (17.9%)	28 (8.9%)	60 (19.2%)	105 (33.5%)
Fish	Pre	9 (3.0%)	48 (15.9%)	83 (27.5%)	44 (14.6%)	17 (5.6%)	44 (14.6%)	57 (18.9%)
	Post	2 (0.6%)	23 (7.4%)	28 (9.0%)	30 (9.6%)	23 (7.4%)	67 (21.5%)	139 (44.6%)
Seafood (Crabs, oyster)	Pre	8 (2.4%)	9 (2.7%)	17 (5.1%)	20 (6.0%)	14 (4.2%)	26 (7.8%)	239 (71.8%)
	Post	4 (1.3%)	7 (2.2%)	6 (1.9%)	10 (3.2%)	8 (2.6%)	26 (8.3%)	252 (80.5%)
Poultry and eggs	Pre	9 (2.9%)	96 (31.0%)	92 (29.7%)	55 (17.7%)	12 (3.9%)	10 (3.2%)	36 (11.6%)
	Post	5 (1.6%)	81 (25.9%)	88 (28.1%)	62 (19.8%)	15 (4.8%)	19 (6.1%)	43 (13.7%)
Pulses and Legumes	Pre	14 (4.5%)	108 (34.5%)	100 (31.9%)	39 (12.5%)	18 (5.8%)	17 (5.4%)	17 (5.4%)
	Post	18 (5.6%)	102 (31.6%)	95 (29.4%)	49 (15.2%)	17 (5.3%)	25 (7.7%)	17 (5.3%)
Nuts and Oilseeds	Pre	10 (3.2%)	60 (19.3%)	75 (24.1%)	67 (21.5%)	25 (8.0%)	44 (14.1%)	30 (9.6%)
	Post	7 (2.3%)	43 (13.9%)	71 (23.0%)	61 (19.7%)	25 (8.1%)	50 (16.2%)	52 (16.8%)
Milk	Pre	46 (14.7%)	110 (35.1%)	70 (22.4%)	37 (11.8%)	11 (3.5%)	10 (3.2%)	29 (9.3%)
	Post	33 (10.6%)	103 (33.0%)	65 (20.8%)	43 (13.8%)	14 (4.5%)	15 (4.8%)	39 (12.5%)
Milk product	Pre	8 (2.6%)	80 (25.6%)	92 (29.4%)	65 (20.8%)	20 (6.4%)	18 (5.8%)	30 (9.6%)
	Post	6 (1.9%)	72 (23.0%)	84 (26.8%)	70 (22.4%)	26 (8.3%)	25 (8.0%)	30 (9.6%)
Vegetable A (Fruit vegetables)	Pre	2 (0.6%)	28 (8.9%)	55 (17.6%)	47 (15.0%)	35 (11.2%)	80 (25.6%)	66 (21.1%)
	Post	1 (0.3%)	20 (6.4%)	48 (15.3%)	52 (16.6%)	36 (11.5%)	87 (27.8%)	69 (22.0%)
Vegetable group B (Roots and tubers)	Pre	0 (0.0%)	5 (1.6%)	35 (11.2%)	65 (20.8%)	40 (12.8%)	100 (31.9%)	68 (21.7%)
	Post	0 (0.0%)	4 (1.3%)	30 (9.5%)	70 (22.1%)	50 (15.8%)	102 (32.2%)	61 (19.2%)
Vegetable group C (Green leafy vegetables)	Pre	3 (1.0%)	33 (10.5%)	75 (24.0%)	60 (19.2%)	28 (8.9%)	80 (25.6%)	34 (10.9%)
	Post	1 (0.3%)	30 (9.5%)	78 (24.7%)	66 (20.9%)	29 (9.2%)	82 (25.9%)	30 (9.5%)
Fruit (common: Apple, banana)	Pre	12 (3.8%)	70 (22.4%)	90 (28.8%)	63 (20.1%)	20 (6.4%)	38 (12.1%)	20 (6.4%)
	Post	10 (3.2%)	66 (21.1%)	88 (28.1%)	65 (20.8%)	24 (7.7%)	40 (12.8%)	36 (11%)
Fruit (Exotic, Kiwi, berries)	Pre	76 (24.3%)	120 (38.3%)	45 (14.4%)	20 (6.4%)	10 (3.2%)	15 (4.8%)	27 (8.6%)
	Post	65 (21.5%)	110 (36.3%)	50 (16.5%)	22 (7.3%)	11 (3.6%)	18 (5.9%)	27 (8.9%)
Sugar	Pre	15 (4.8%)	66 (21.1%)	83 (26.5%)	52 (16.6%)	23 (7.3%)	42 (13.4%)	32 (10.2%)
	Post	11 (3.6%)	60 (19.5%)	80 (26.0%)	54 (17.5%)	25 (8.1%)	45 (14.6%)	33 (10.7%)
Fat/oils	Pre	5 (1.6%)	30 (9.6%)	55 (17.6%)	60 (19.2%)	40 (12.8%)	77 (24.6%)	46 (14.7%)
	Post	3 (1.0%)	25 (8.0%)	50 (16.0%)	63 (20.1%)	45 (14.4%)	80 (25.6%)	47 (15.0%)
Convenience food /processed food	Pre	10 (3.2%)	60 (19.2%)	65 (20.8%)	56 (17.9%)	34 (10.9%)	43 (13.7%)	45 (14.4%)
	Post	9 (2.9%)	55 (17.7%)	62 (20.0%)	58 (18.7%)	33 (10.6%)	47 (15.2%)	46 (14.8%)
Dietary supplements, including herbal	Pre	1 (0.3%)	5 (1.6%)	30 (9.6%)	35 (11.2%)	28 (8.9%)	60 (19.2%)	154 (49.2%)
	Post	1 (0.3%)	4 (1.3%)	28 (8.9%)	33 (10.5%)	27 (8.6%)	65 (20.8%)	155 (49.5%)

Table 2: Conversion rules for the calculation of the daily amount consumed by International Students

Frequency	Conversion formula
Twice daily	Total daily amount*2
2-3 times a week	Total weekly amount* 2.5 (avg frequency)/ 7 days
Weekly	Total weekly amount/7days
Twice monthly	Total monthly amount*2/30 days
Monthly	Total monthly amount/ 30 days
